# Supplementary material for: Concurrent tau pathologies in frontotemporal lobar degeneration with TDP‐43 pathology
Source: Neuropathol Appl Neurobiol. 2021 Dec 10;48(2):e12778. doi: 10.1111/nan.12778 (PMC9300011; doi:10.1111/nan.12778)
Supplement: Supplementary file 1 — Data S1. Supporting Information [file NAN-48-0-s001.docx]

**SUPPLEMENTARY INFORMATION**

*Concurrent tau pathologies in frontotemporal lobar degeneration with TDP-43*

Shunsuke Koga, MD, PhD, Xiaolai Zhou, MD, PhD, Aya Murakami, MD, PhD, Cristhoper Fernandez De Castro, Matthew C. Baker, Rosa Rademakers, PhD, and Dennis W. Dickson, MD

**Contents**

Supplementary Appendix1

Supplementary Data2

**Supplementary Data: Clinical history of two patients with FTLD-TDP and CBD**

Case 1

This patient was an 89-year-old, right-handed Hispanic man with an 8-year history of memory impairment and a 3-year history of disorientation, socially inappropriate behavior, and apathy. His family history was notable for late-onset (70s to 80s) dementia in his father and two siblings who died in the 80s to 90s. Neurological examination revealed saccadic eye movements, action tremor in both upper extremities, mild bradykinesia in both lower extremities, and a slow unsteady gait. He scored 9/30 on the Mini-Mental State Examination. He was diagnosed with familial Alzheimer's disease. His dementia and gait difficulty gradually worsened. He developed frequent falls in the last 6 months of his life.

Case 2

This 84-year-old Caucasian man had a six-year history of memory loss and behavior change, characterized by agitation, mutism and violent outbursts, treated with rivastigmine, memantine, and risperidone. He had no family history of neurological disorders. On neurological examination at 78 years of age, he scored 15/30 on the Mini-Mental State Examination. An MRI of the brain revealed mild cortical atrophy, and FDG-PET revealed decreased glucose metabolism in the bilateral frontal and temporal lobes. He was diagnosed with frontotemporal dementia with a differential diagnosis of mixed dementia (i.e., Alzheimer’s-type, vascular, and alcohol-related dementia)
